# ﻿A new species of the genus *Soriculus* (Soricidae, Eulipotyphla, Mammalia) from Medog, Tibet, China, based on morphological and molecular data

**DOI:** 10.3897/zookeys.1262.164459

**Published:** 2025-12-04

**Authors:** Tao Zhang, Siyu Yang, Haijun Jiang, Lin Gu, Qingfang Zou, Changkun Fu, Keji Guo, Tong Zhang, Shaoying Liu, Shunde Chen

**Affiliations:** 1 College of Life Sciences, Sichuan Normal University, Chengdu 610066, China; 2 School of Life Sciences, Zhengzhou University, Zhengzhou, China; 3 Institute of Biodiversity and Ecology, Zhengzhou University, Zhengzhou, China; 4 Kunming Institute of Zoology, Chinese Academy of Sciences, Kunming 650201, China; 5 Central South Inventory and Planning Institute of National Forestry and Grassland Administration, Changsha, Hunan 410014, China; 6 Sichuan Academy of Forestry, Chengdu 610081, China

**Keywords:** Morphology, new species, phylogeny, shrew, small mammals, *
Soriculus
*, taxonomy

## Abstract

The genus *Soriculus* (Soricidae, Eulipotyphla) currently comprises five recognized species, predominantly distributed in the Himalayas and the Gaoligong Mountains. During our survey of small mammals in Medog County, Tibet, China, 11 *Soriculus* specimens were collected. In this study, we conducted phylogenetic analyses of the genus using one mitochondrial gene (*Cytb*) and three nuclear genes (*BRCA1*, *APOB*, and *RAG2*) to assess the phylogenetic relationships of these specimens. The morphology of the 11 specimens was compared with other species within the genus. Our results demonstrate that these specimens represent a new species, *Soriculus dexingensis***sp. nov.** Phylogenetic analyses revealed that *S. dexingensis***sp. nov.** forms a distinct sister clade to *S. medogensis*, and the Kimura 2-Parameter (K2P) distances between all known species of *Soriculus* ranged from 0.111 to 0.187, indicating interspecific divergence. Morphologically, the new species is distinguished by a relatively longer tail and a significantly higher TL/HB ratio. The discovery of *S. dexingensis***sp. nov.** in Medog County suggests that the diversity of *Soriculus* species remains underestimated. Further biodiversity surveys of small mammals across the Himalayan region are warranted.

## ﻿Introduction

The genus *Soriculus* Blyth, 1854 comprises shrews primarily distributed at moderate to high elevations in the Himalayan region (Hutterer 2005; Motokawa et al. 2008). Members of the genus possess enlarged foreclaws, likely adapted for digging and fossorial habits (Hoffman 1986). Initially, the genus *Soriculus* was considered to include two subgenera: *Episoriculus* and *Chodsigoa* (Ellerman and Morrison-Scott 1951; Hoffman 1986; Motokawa 2003). However, these three taxa were subsequently recognized as distinct genera based on morphological and molecular evidence (Repenning 1967; Hutterer 2005; He et al. 2010). Despite these revisions, the diversity of *Soriculus* has long been underestimated. For decades, *Soriculus* was regarded as monotypic, containing only one species (*Soriculus nigrescens* Gray, 1840) and two subspecies (*S. n. nigrescens* and *S. n. minor* Dobson, 1890). This classification was widely accepted by scholars (Smith and Xie 2009; Burgin and He 2018).

The divergence of *Soriculus* was first revealed by Jiang et al. (2023). Their study supported the classification of the genus *Soriculus* into three evolutionary clades, but morphological evidence was not incorporated to further clarify the phylogenetic relationships within the genus. Subsequently, Chen et al. (2024) conducted a comprehensive integrated analysis of the genus, elevating *S. n. minor* to species status. Furthermore, they described two new species: *Soriculus nivatus* (Chen & Jiang, 2024) and *Soriculus medogensis* (Chen & Jiang, 2024). They also identified a new species (known from a single specimen), which was later described as *Soriculus beibengensis* (Pei et al. 2024) based on nine additional specimens from Medog, following molecular and morphological analyses (Pei et al. 2024).

During fieldwork in 2011, we collected 11 *Soriculus* specimens from Medog, Tibet, China. These specimens were previously assigned to *S. medogensis* (Chen et al. 2024; Pei et al. 2024). However, recent integrated molecular and morphological analyses have revealed substantial genetic divergence from known congeners. They are therefore recognized as a putative new species (referred to as *Soriculus* sp. in the Material and methods and Results sections).

## ﻿Material and methods

### ﻿Ethics statement

All specimens were obtained in accordance with the guidelines of the American Society of Mammalogists and the laws and regulations of China concerning the protection of wild terrestrial animals (State Council Decree 1992; Sikes and Animal 2016), and the Guidelines for Care and Use of Laboratory Animals at the Ethics Committee at Sichuan Normal University (Chengdu, China).

### ﻿Samples and sequencing

In October 2011, a total of eleven individuals (*Soriculus* sp.) were collected using snap traps from Medog, Tibet, China (Suppl. material 1: table S1). Fresh muscle and liver tissues were taken from each individual, immediately preserved in 95% ethanol, and stored at -80 °C for molecular analyses. Tissue samples and all specimens used in this study are deposited in Sichuan Normal University (SCNU) and the Sichuan Academy of Forestry (SAF).

Total DNA was extracted from muscle or liver tissues using an animal tissue DNA extraction kit (Chengdu Fuji Biotechnology Co., Ltd, Sichuan, China). One mitochondrial gene (*Cytb*, 1140 bp) and three nuclear genes [apolipoprotein B (*APOB*), breast cancer 1 (*BRCA1*), and recombination activating protein 2 (*RAG2*)] were amplified. The primers used and the PCR program conditions were the same as those described in Jiang et al. (2023). All DNA sequences were edited with EditSeq (DNASTAR, Lasergene v. 7.1) and further aligned in MEGA 11 (Tamura et al. 2021).

Corresponding sequences of published *Soriculus* individuals and sequences of other soricid genera were obtained from GenBank (Suppl. material 1: table S2).

### ﻿Phylogenetic analysis

Two datasets were constructed for phylogenetic analysis: (1) a dataset of the mitochondrial gene (mtDNA); (2) a dataset of concatenated nuclear genes (nDNA). MrBayes v. 3.2.7 (Ronquist et al. 2012) was used for the Bayesian inference analysis. Each run was performed using four Markov chain Monte Carlo (MCMC) algorithms, with 10,000,000 generations for both the single-gene dataset and the concatenated gene datasets. IQ-TREE v. 3.0.1 (Nguyen et al. 2015) was used for maximum likelihood (ML) analyses, with 10,000 ultrafast bootstraps to estimate branch support. ModelFinder (in IQ-TREE) was used to determine the optimal model for each gene, and the fitness of the model was estimated by the Akaike Information Criterion (AIC) (Luo et al. 2010). All the above analyses were conducted in PhyloSuite v. 1.2.3 (Zhang et al. 2020). Posterior probabilities (PP) > 95% and ultrafast bootstrap values (UFBoot) ≥ 95 were considered strongly supported (Huelsenbeck and Rannala 2004; Minh et al. 2018).

### ﻿Genetic distances and species delimitation

The Kimura-2-parameter (K2P) distances for species/putative species based on the *Cytb* gene were calculated in MEGA 11 (Kimura 1980; Tamura et al. 2021).

Bayesian Phylogenetics and Phylogeography (BPP) analyses were performed using the nDNA dataset and the combined mtDNA+nDNA dataset in BPP v. 3.1 (Yang and Rannala 2010). Following Chen et al. (2022), we used two alternative rjMCMC algorithms (algorithms 0 and 1). The ancestral population size and root age were represented by θ and τ, respectively. The three combination priors on θ and τ were adopted: (1) G (1, 10) for θ and G (1, 10) for τ; (2) G (1, 10) for θ and G (2, 2000) for τ; and (3) G (2, 2000) for θ and G (2, 2000) for τ. Each rjMCMC was run for 100,000 generations, with samples collected every 100 generations after discarding 10,000 generations as pre-burn-in. Each clade was supported as an independent species if the posterior probability was greater than 0.95.

### ﻿Divergence time

We used the nDNA dataset to estimate the divergence times in BEAST v. 2.6 (Bouckaert R et al. 2014). Two fossil calibration points were used following Chen et al. (2024): (1) The split between Crocidurinae and Soricinae was approximately 36 Ma (Springer et al. 2018). The offset was set to 0 and the mean to 36, with a standard deviation of 0.135; and (2) The first fossil record of Blarinellini was from the Early Middle Miocene (Harris 1998; Rzebik-Kowalska 1998), and the oldest divergence of Blarinini occurred in the Barstovian (13.6–16.3 Ma) (Repenning 1967). We set the offset to 15, the mean to 0, and the standard deviation to 0.98. The BEAST analysis used a Birth-Death tree prior and a relaxed lognormal clock model. Each analysis ran for 100 million generations and was sampled every 5000 generations. The posterior distributions and ESSs of each parameter greater than 200 was calculated in Tracer v. 1.7 (Rambaut et al. 2018). TreeAnnotator v. 1.6.1 was used to determine the burn-in fraction, set to the first 25% of the generations.

### ﻿Morphological analysis

A total of 62 preserved specimens of *Soriculus*, including *S. beibengensis* (*N* = 8), *S. nigrescens* (*N* = 14), *S. medogensis* (*N* = 4), *S. nivatus* (*N* = 20), *S. minor* (*N* = 5), and 11 specimens of *Soriculus* sp. were used for morphological analysis. The external measurements of these specimens were taken in the field, including weight (W), head-body length (HBL), tail length (TL), hind foot length (HL), and ear length (EL). Eleven craniodental metrics were measured by a digital caliper graduated to 0.01 mm following Pan et al. (2007) and Yang et al. (2007). The morphological characters of the skull and their abbreviations are: condyle-incisive length (CIL), braincase height (BH), interorbital breadth (IOB), rostral breadth (RB), braincase breadth (BB), upper toothrow length (UTR), palatoincisive length (PIL), postpalatal length (PPL), maximum width across the upper second molars (M^2^–M^2^), mandibular length (ML), lower toothrow length (LTR). All craniodental measurements were taken by Siyu Yang, except for measurements of five *S. minor* specimens provided by the Kunming Institute of Zoology (KIZ). Measured specimens were listed in the Suppl. material 1: table S3.

The measurements of the skull were analyzed using SPSS v. 26.0 (SPSS, Chicago, IL, USA) for principal component analysis (PCA) and canonical discriminant function analysis (DFA). The terminologies for morphological descriptions followed Motokawa and Lin (2005) and Chen et al. (2024).

## ﻿Results

### ﻿Morphological analysis

**Figure 1.**
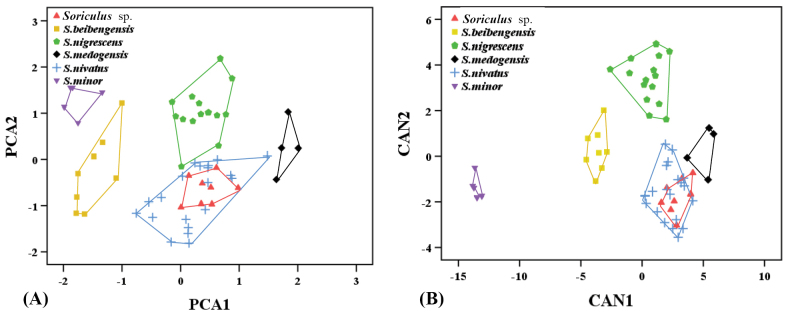
Results of principal component analysis (A) and discriminant function analysis (B) based on the skull measurements of the genus *Soriculus*.

The external (*N* = 62) and craniodental (*N* = 58) measurements of *Soriculus* are given in Table 1. Results of the Kaiser–Meyer–Olkin measure of sampling adequacy indicated that the data were suitable for PCA (KMO = 0.904, Bartlett’s test < 0.001). In the PCA analysis, two principal components were extracted, which explained 87.41% of the total variance (Table 2). The eigenvalues of two PCs exceeded 1.0. PC1 explained 75.27% of the variation, and all factor loadings were positive. The factor loadings of CIL, PIL, UTR and LTR were greater than 0.9, and the factor loadings of PPL, IOB, BB, BH and ML were greater than 0.8. PC2 explained 12.14% of the variance and was negatively correlated with most variables except IOB, RB, M^2^M^2^, BB and BH. PCA scatterplots (Fig. 1A) showed that different species in *Soriculus* exhibited a clear separation trend. However, *S. nivatus* and *Soriculus* sp. overlapped to a high degree. *Soriculus* sp. specimens occupied the positive region of PC1 and the negative region of PC2, indicating that this new species had a larger and narrower skull than other recognized species, but smaller than *S. medogensis*. The results of the DFA correctly classified 89.8% of the specimens. One individual of *Soriculus* sp. was assigned to *S. nivatus.* Four *S. nivatus* individuals were assigned to *Soriculus* sp., and one *S. nivatus* individual was assigned to *S. medogensis*. Plots of CAN1 and CAN2 (Fig. 1B) showed that most species separated well, but *S. nivatus* and *Soriculus* sp. still could not be separated from each other.

**Table 1.** Means (mm), standard deviation and ranges of the measurement data of the skull and external morphology for different species of *Soriculus* used in this study.

Variable	*S. nigrescens*	*S. nivatus*	*S. medogensis*	*S. beibengensis*	*S. minor*	*Soriculus* sp.
	*N* = 14	*N* = 20	*N* = 4	*N* = 8	*N* = 5	*N* = 11
W	16.7 ± 1.7	13.1 ± 2.8	15.8 ± 1.4	10.0 ± 2.0	9.3 ± 1.0	12.7 ± 1.2
	13.0–19.0; 14	7.0–19.0; 20	14.5–18.0; 4	7.0–12.3; 8	8.2–10.7; 5	11.7–15.3; 11
HBL	87 ± 3	80 ± 5	82 ± 3	71 ± 6	68 ± 4	78 ± 4
	82–92; 14	73–89; 19	77–84; 4	59–77; 8	62–71; 5	71–84; 11
TL	42 ± 2	49 ± 5	54 ± 2	40 ± 3	38 ± 4	57 ± 3
	38–46; 14	40–57; 20	52–56; 4	34–43; 6	32–43; 4	50–62; 11
HL	15 ± 0	15 ± 1	16 ± 1	13 ± 1	12.4 ± 1	15 ± 0
	14–16; 14	13–15.5; 20	15–16; 4	12–15; 8	12–14; 5	15; 11
EL	5 ± 0	7 ± 1	7 ± 2	4 ± 1	8 ± 1	7 ± 0
	5; 7	5–10; 12	4–8; 4	3–6; 8	7–10; 5	7; 11
CIL	22.07 ± 0.39	22.69 ± 0.49	23.79 ± 0.24	20.27 ± 0.18	19.63 ± 0.39	22.87 ± 0.38
	21.43–22.68; 14	21.71–23.92; 20	23.62–24.01; 4	20.03–20.68; 8	19.18–20.22; 5	22.20–23.54; 6
PIL	10.11 ± 0.19	10.24 ± 0.34	11.19 ± 0.26	9.09 ± 0.14	8.86 ± 0.27	10.52 ± 0.32
	9.82–10.50; 14	9.53–10.82; 20	10.82–11.38; 4	8.87–9.26; 8	8.6–9.28; 5	9.94–11.01; 8
PPL	9.62 ± 0.20	9.7 ± 0.3	10.14 ± 0.26	9.01 ± 0.12	8.76 ± 0.13	9.69 ± 0.19
	9.29–9.99; 14	9.04–10.24; 20	9.8–10.42; 4	8.86–9.25; 7	8.58–8.94; 5	9.37–10.06; 7
UTR	9.95 ± 0.17	10.03 ± 0.31	10.92 ± 0.17	8.91 ± 0.13	8.51 ± 0.23	10.20 ± 0.16
	9.57–10.17; 14	9.36–10.51; 20	10.78–11.11; 4	8.78–9.17; 8	8.29–8.89; 5	9.89–10.38; 8
IOB	5.24 ± 0.17	4.96 ± 0.17	5.3 ± 0.08	4.55 ± 0.16	4.78 ± 0.12	5.00 ± 0.12
	4.87–5.48; 14	4.75–5.46; 20	5.21–5.41; 4	4.35–4.87; 8	4.67–4.96; 5	4.78–5.13; 8
RB	6.35 ± 0.17	6.03 ± 0.25	6.65 ± 0.25	5.82 ± 0.27	6 ± 0.13	6.10 ± 0.18
	6.13–6.72; 14	5.64–6.48; 20	6.38–6.55; 4	5.44–6.25; 8	5.79–6.12; 5	5.82–6.32; 8
M^2^M^2^	6.46 ± 0.16	6 ± 0.3	6.66 ± 0.19	5.65 ± 0.25	5.67 ± 0.07	6.01 ± 0.09
	6.19–6.82; 14	5.36–6.37; 20	6.41–6.83; 4	5.30–6.04; 8	5.57–5.75; 5	5.90–6.12; 8
BB	11.16 ± 0.31	11.05 ± 0.39	11.93 ± 0.12	10.43 ± 0.28	10.56 ± 0.16	11.37 ± 0.17
	10.52–11.72; 14	9.99–11.79; 19	11.78–12.06; 4	9.99–10.75; 8	10.38–10.71; 5	11.16–11.68; 8
BH	6.65 ± 0.13	6.55 ± 0.19	6.91 ± 0.36	6.04 ± 0.25	6.20 ± 0.24	6.56 ± 0.13
	6.35–6.82; 14	6.08–6.9; 20	6.39–7.2; 4	5.69–6.54; 8	5.94–6.57; 5	6.38–6.75; 7
ML	13.90 ± 0.29	14.29 ± 0.37	15.42 ± 0.22	12.73 ± 0.15	10.85 ± 0.11	14.51 ± 0.28
	13.14–14.25; 14	13.28–15.01; 20	15.21–15.62; 4	12.43–12.93; 8	10.68–10.99; 5	14.20–14.96; 8
LTR	8.89 ± 0.26	9.18 ± 0.35	9.99 ± 0.08	8.12 ± 0.13	7.78 ± 0.20	9.23 ± 0.16
	8.42–9.35; 14	7.99–9.7; 20	9.89–10.09; 4	7.92–8.27; 8	7.65–8.11; 5	8.91–9.39; 8

**Table 2.** Factor loadings, eigenvalues, and total variance explained by the two principal components of the PCA of 11 craniodental measurements of 58 specimens of *Soriculus*.

Variables	Component	
	1	2
CIL	0.938	-0.298
PIL	0.960	-0.185
PPL	0.882	-0.210
UTR	0.953	-0.217
IOB	0.806	0.446
RB	0.706	0.619
M2–M2	0.772	0.551
BB	0.858	0.101
BH	0.822	0.139
ML	0.895	-0.363
LTR	0.913	-0.273
Eigenvalue	8.280	1.335
Total variance explained (%)	75.273	12.142

### ﻿Phylogenetic analyses

*Cytb* and nuclear genes from all 11 specimens of the new species were obtained: 1140 bp for the mitochondrial gene and 1894 bp for the nuclear genes (APOB, BRCA1, and RAG2). Sequences generated for the new species were deposited in GenBank (Suppl. material 1: table S1).

**Figure 2.**
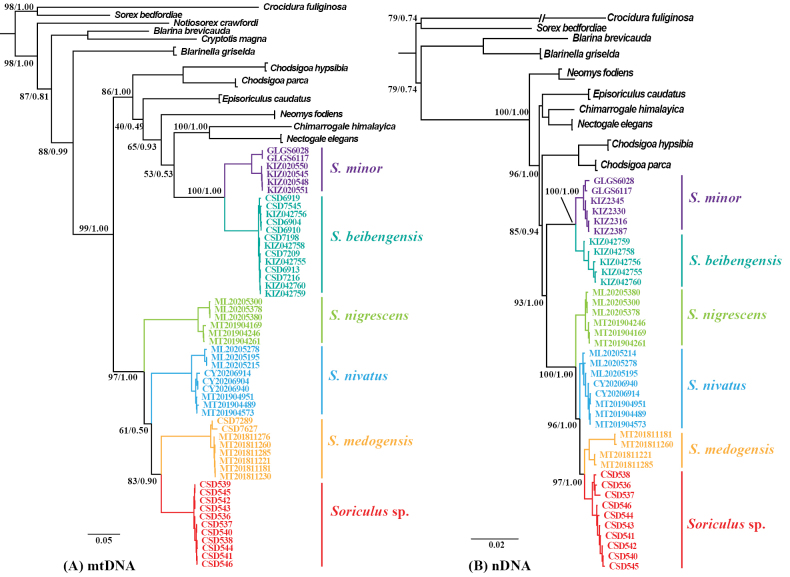
Phylogenetic trees of the genus *Soriculus* based on the mtDNA dataset and the concatenated nDNA dataset. Node numbers indicate ultrafast bootstrap values (left) and Bayesian posterior probabilities (right).

Phylogenetic trees reconstructed using Bayesian inference and maximum likelihood analyses based on two datasets (mtDNA and nuDNA) exhibited similar topological structures (only the BI tree is shown in Fig. 2). The phylogenetic trees generated from two datasets both supported the classification of these 11 specimens into a new species. Furthermore, the phylogenetic trees based on nDNA strongly supported the monophyly of *Soriculus*, which was divided into two primary clades: Clade I comprised *S. minor* and *S. beibengensis*; Clade II consisted of *S. nigrescens*, *Soriculus* sp., *S. medogensis*, and *S. nivatus*. Within Clade II, *Soriculus* sp. first formed a distinct branch, which then grouped as a sister clade to *S. medogensis* with high statistical support (PP = 1.00, UFBoot = 97). The mtDNA tree also showed a sister relationship between *Soriculus* sp. and *S. medogensis* (PP = 0.90, UFBoot = 83). However, the support for this relationship between *S. nivatus* and (*Soriculus* sp. + *S. medogensis*) was low (PP = 0.50, UFBoot = 61).

### ﻿Genetic distances and species delimitation

The Kimura-2-parameter (K2P) genetic distances of the *Cytb* gene among species in the genus *Soriculus* ranged from 0.111 to 0.187. The genetic distances between *Soriculus* sp. and other species ranged from 0.111 (with *S. nivatus* and *S. medogensis*) to 0.181 (with *S. beibengensis*). Genetic distances among species were consistent with species-level divergence (Table 3).

**Table 3.** Kimura two-parameters genetic distances of *Soriculus* based on the *Cytb* gene.

	*S. minor*	*S. beibengensis*	*S. nigrescens*	*S. nivatus*	*S. medogensis*
*S. beibengensis*	0.100				
*S. nigrescens*	0.187	0.175			
*S. nivatus*	0.170	0.169	0.131		
*S. medogensis*	0.184	0.189	0.145	0.122	
*Soriculus* sp.	0.177	0.181	0.118	0.111	0.111

The BPP results based on the mtDNA + nuDNA and nuDNA datasets produced 36 outcomes, all of which strongly supported six species (PP > 0.99; Suppl. material 1: table S4).

### ﻿Molecular divergence estimation

**Figure 3.**
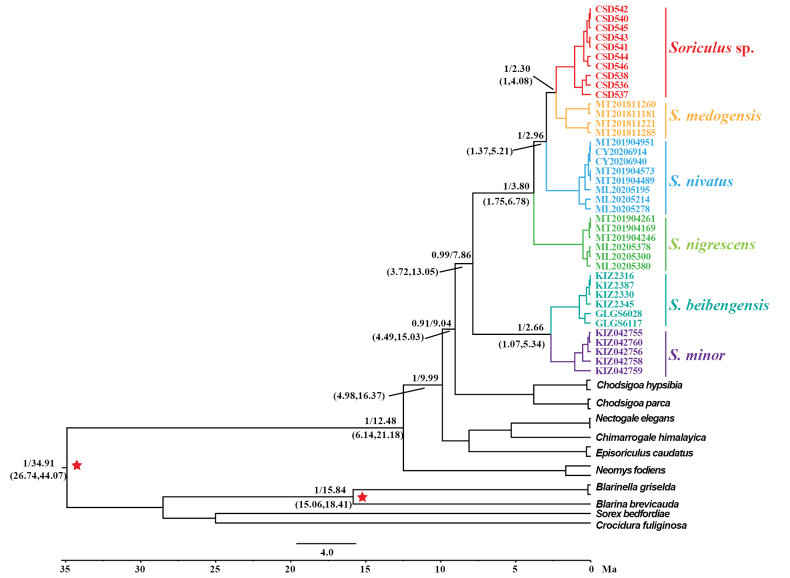
Divergence times estimated using BEAST based on the nDNA dataset. Branch lengths represent time (Ma). The two red asterisks indicate fossil-calibrated nodes. Numbers left of the slash represent the posterior probabilities (PP) of each node, numbers right of the slash represent the median divergence time, and numbers within parentheses indicate the confidence interval.

The topological structure of the divergence time tree based on nDNA was the same as that of the nDNA tree (Fig. 3). The results showed that the latest common ancestor of *Soriculus* can be traced back to the Late Miocene (7.86 Ma, 95% CI = 3.72–15.03). Both the divergence time of *S. nivatus* and (*S. medogensis* + *Soriculus* sp.) (2.96 Ma, 95% CI = 1.37–5.21) and the divergence time of *Soriculus* sp. and *S. medogensis* (2.3 Ma, 95% CI =1–4.08) occurred in the Early Pleistocene.

## ﻿Taxonomy

### ﻿Order Eulipotyphla Waddell et al., 1999

**Family Soricidae G. Fischer, 1814**

**Subfamily Soricinae G. Fischer, 1814**

**Tribe Nectogalini Anderson, 1879**

**Genus *Soriculus* Blyth, 1854**

#### ﻿ Soriculus dexingensis

Taxon classificationAnimaliaEulipotyphlaSoricidae

Zhang, Liu & Chen sp. nov.

4A580EBF-183E-5B72-9588-B8133D1403AE

https://zoobank.org/EA040A43-90E1-4132-937B-F754909E6E32

##### Holotype.

SAF11216, adult male, collected on 29 October 2011 by Rui Liao. The specimen was deposited at the Sichuan Academy of Forestry (SAF).

##### Measurements of holotype (mm).

W = 12.1 g, HB = 80, TL = 59, HL = 15, EL = 7, CIL = 23.54, PIL = 11.01, PPL = 10.06, UTR = 10.38, IOB = 5.13, RB = 6.32, M^2^M^2^ = 6.11, BB = 11.23, BH = 6.75, ML = 14.96, LTR = 9.39.

##### Type locality.

Dexing Town, Medog County, Tibet, China (29.41778°N, 95.05969°E, 2100 m a.s.l.).

##### Paratypes.

Seven specimens SAF11200 (male), SAF11215 (male), SAF11237 (female), SAF11238 (male), SAF11243 (male), SAF11245 (male), SAF11246 (male). Collected from the type locality in Medog in October 2011 at elevations from 2100 m to 2832 m. All specimens are deposited in SAF.

##### Diagnosis.

Size similar to *S. nivatus*, much larger than *S. minor* and *S. beibengensis*, but smaller than *S. nigrescens* and *S. medogensis*. The tail averages 74% of the head and body length, the longest within *Soriculus*. The teeth are more robust than those in *S. nivatus*. The maximum width across the upper second molars (M^2^M^2^) is less than that of *S. medogensis* and *S. nigrescens.* The pigmentation of the teeth is much heavier than that in *S. minor* and *S. beibengensis*.

##### Description.

**Figure 4.**
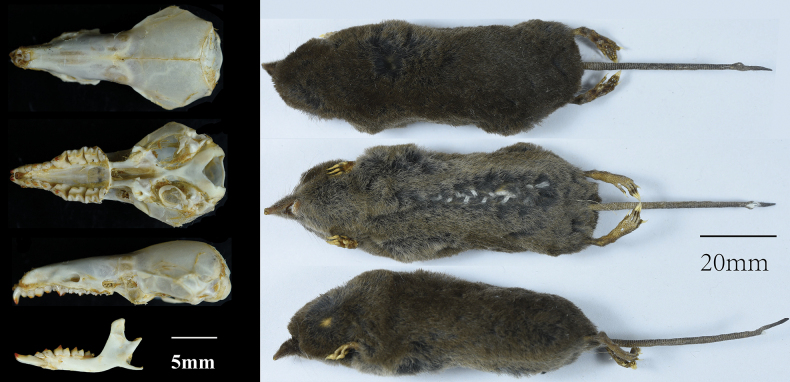
Left: Dorsal, ventral and lateral views of the skull and mandibles of *S. dexingensis* sp. nov. (SAF11216). Right: dorsal, ventral, and lateral views of *S. dexingensis* sp. nov. (SAF11216).

*Soriculus dexingensis* sp. nov. is a medium-sized shrew in the genus *Soriculus* (W=12.74 ± 1.20 g, BH=77.81 ± 4.28 mm). The dorsal hair is brownish with a blackish-gray base and dark brown tip. The ventral hair is dark gray, not distinctly different from the dorsal hair (Fig. 4). The tail is bicolored: dorsal part dark brown; ventral part slightly lighter (Fig. 4). The tail is relatively long (nine of eleven specimens with TL ≥ 56 mm, 57.45 ± 3.47 mm), averaging 74% of the head and body length (HB). The foreclaws are enlarged. The dorsal surfaces of the hands and feet are covered with short, dark brown hairs.

The skull is relatively large within the genus *Soriculus*, but smaller than that of *S. medogensis*. Braincase is domed but low, and the posterior of the skull is flattened. The sagittal and lambdoidal crests are moderately developed. The rostrum is relatively low, and the maxillary region is narrow. The basioccipital is slender, fused with the basisphenoid in the central region. The coronoid process of the mandible is robust and long, with a spatulate tip that rises straight upward. The condyloid process forms an angle of roughly 45° with the coronoid process. The angular process is long and thin, slightly bent, with an expanded, upwardly bent tip.

The dental formula of *S. dexingensis* sp. nov. is: I 3/2, C 1/0, P 2/1, M 3/3 (×2) = 30, which is consistent with that of the genus *Soriculus*. The teeth are robust. The apex of the first upper incisor points straight downward, with a broad posterior cusp. Four upper unicuspids (U^1^–U^4^) are present: U^2^ is the largest in size, U^1^ is slightly smaller and similar to U^3^, and U^4^ is minute. M^1^ and M^2^ are similar in size, whereas M^3^ is significantly reduced. The lower incisor (I_1_) is straight and long, with a low cusp. Half of the lower unicuspid (U_1_) is in contact with I_1_. M_1_ is larger than M_2_, and M_3_ is the smallest. The tips of all teeth are pigmented red-orange.

##### Suggested common name.

Dexing Large-clawed Shrew (English), 德兴大爪鼩鼱(Chinese).

##### Etymology.

The specific Latin name *dexingensis* is named for Dexing, the type locality, with the Latin adjectival suffix –ensis meaning “belonging to”.

##### Comparison.

Morphologically, *Soriculus dexingensis* sp. nov. is similar to *S. nivatus*, but can be distinguished from the latter by many characters. The tail of *S. dexingensis* sp. nov. is relatively longer (TL = 57.45 ± 3.31 mm) than that of *S. nivatus* (TL = 49.38 ± 5.05 mm); nine of eleven *S. dexingensis* sp. nov. individuals have a tail length greater than 56 mm, whereas most individuals of *S. nivatus* (18 of 20) have a tail length less than 56 mm. The TL/HB ratio of *S. dexingensis* sp. nov. (74%) is much larger than that of *S. nivatus* (64%). In the skull, *S. dexingensis* sp. nov. is similar to *S. nivatus* and cannot be distinguished from it by skull measurements. However, the teeth of *S. nivatus* are much more slender and delicate than those of *S. dexingensis* sp. nov. The posterior of the skull of *S. dexingensis* sp. nov. is flattened, whereas that of *S. nivatus* is rounded. In addition, *S. dexingensis* sp. nov. has more lightly pigmented teeth than those of *S. nivatus*.

*Soriculus dexingensis* sp. nov. can be easily distinguished from *S. minor* and *S. beibengensis* by its larger size, and all measurements of *S. dexingensis* sp. nov. show nearly no overlap with measurements of *S. minor* and *S. beibengensis* (Table 1). Furthermore, it can be distinguished by its longer tail (TL/HB ratio: 56% in *S. minor* and 56% in *S. beibengensis*) and by its distinctly more heavily pigmented teeth compared to these two species.

*Soriculus dexingensis* sp. nov. can also be distinguished from *S. nigrescens* by its longer tail (TL = 57 ± 3 mm, range 50–62 mm vs. TL = 42 ± 2 mm, range 38–46 mm in *S. nigrescens*). Besides, the rostrum of *S. dexingensis* sp. nov. is relatively narrower than that of *S. nigrescens*, and the range of M^2^M^2^ (5.90–6.12 mm in *S. dexingensis* sp. nov. vs. 6.19–6.82 mm in *S. nigrescens*) does not overlap between the two species.

Compared to its sister species *S. medogensis*, *S. dexingensis* sp. nov. can be distinguished by its smaller skull and more heavily pigmented teeth. Except for two skull measurements (PPL, PIL), all other measurements of *S. dexingensis* sp. nov. are smaller than those of *S. medogensis* with no overlap. Additionally, the fourth upper unicuspid (U^4^) of *S. dexingensis* sp. nov. is smaller than that in *S. medogensis*.

##### Comments.

Specimens (CSD536–CSD546) previously assigned to *Soriculus medogensis* by Chen et al. (2024) were re-examined using an integrative approach combining morphological and molecular data. Our analyses reveal that these individuals exhibit substantial genetic divergence and significant morphological differences from all known *Soriculus* species. Consequently, we describe them herein as a new species.

##### Distribution.

*Soriculus dexingensis* sp. nov. is currently only known from elevations of 2100–2830 m in the eastern Himalayas, specifically in Medog County, Tibet, China. The specimens were all captured from broadleaf forests.

## ﻿Discussion

*Soriculus* was previously recognized as a monotypic genus (Hutterer 2005; Burgin and He 2018). However, based on integrating molecular and morphological analyses, Chen et al. (2024) revealed the diversity of the genus *Soriculus*. Currently, five species of *Soriculus* have been described. In this study, we describe another new species, *S. dexingensis* sp. nov., collected in Medog, Tibet, China. It is worth noting that the genus is most diverse in Medog County, Tibet, with five species present (*S. nigrescens*, *S. medogensis*, *S. nivatus*, *S. beibengensis*, and *Soriculus dexingensis* sp. nov.) (Suppl. material 2). Additionally, *S. medogensis* was sympatric with *S. nivatus* (Chen et al. 2024). Under the influence of global cooling and desiccation, the genus *Soriculus* likely migrated southwards and adopted Medog as a key refuge (Pei et al. 2024). We hypothesize that the fossorial habits of *Soriculus*, together with the complex topography and habitat of Medog County, may have promoted speciation by limiting interspecific communication.

The eastern Himalayas is among the most biologically diverse regions in the world (Lily et al. 2022). This study expands our understanding of the diversity within *Soriculus* in the eastern Himalayas. Furthermore, previous molecular analyses have shown that specimens of *S. nigrescens* from Dingri, Tibet, China (csd584, Jiang et al. 2023) are deeply divergent from other *S. nigrescens* specimens (Chen et al. 2024), suggesting that species diversity within the genus *Soriculus* remains underestimated in southern Tibet. Indeed, new species of small mammals have been recently discovered and described from this region (Wang et al. 2025). Therefore, enhanced biodiversity surveys of small mammals across the Himalayan region will likely clarify the dispersal history of *Soriculus* and reveal additional overlooked biodiversity.

## Supplementary Material

XML Treatment for Soriculus dexingensis

## ﻿Additional information

### Use of AI

No use of AI was reported.

### Author ORCIDs

Tao Zhang https://orcid.org/0009-0006-8077-9502

Tong Zhang https://orcid.org/0009-0009-1492-585X

Shaoying Liu https://orcid.org/0000-0003-1382-7149

### Data availability

All of the data that support the findings of this study are available in the main text or Supplementary Information.

## Supplementary materials

Supplementary material 1 Supplementary tables Data type xlsx Explanation note table S1. Information regarding new species and samples with newly generated sequences; table S2. Sample information and sequence data for the additional taxa included in the molecular analysis; table S3. External and Cranial measurements of the genus Soriculus used in this study; table S4. Posterior probabilities for 6 species of Soriculus by using different priors for model parameters.. https://binary.pensoft.net/file/1484449This dataset is made available under the Open Database License (http://opendatacommons.org/licenses/odbl/1.0/). The Open Database License (ODbL) is a license agreement intended to allow users to freely share, modify, and use this Dataset while maintaining this same freedom for others, provided that the original source and author(s) are credited.Tao Zhang, Siyu Yang, Haijun Jiang, lin Gu, Qingfang Zou, Changkun Fu, Keji Guo, Tong Zhang, Shaoying Liu, Shunde Chen

Supplementary material 2 Supplementary figure Data type docx Explanation note Sample localities for the phylogenetic analysis include the putative species (described by Chen et al. 2024), which refers to the *Soriculus* specimens from Dingri, Tibet, China (voucher csd584; Jiang et al. 2023). https://binary.pensoft.net/file/1484450This dataset is made available under the Open Database License (http://opendatacommons.org/licenses/odbl/1.0/). The Open Database License (ODbL) is a license agreement intended to allow users to freely share, modify, and use this Dataset while maintaining this same freedom for others, provided that the original source and author(s) are credited.Tao Zhang, Siyu Yang, Haijun Jiang, lin Gu, Qingfang Zou, Changkun Fu, Keji Guo, Tong Zhang, Shaoying Liu, Shunde Chen
