# Supplementary material for: ﻿A new species of the genus Soriculus (Soricidae, Eulipotyphla, Mammalia) from Medog, Tibet, China, based on morphological and molecular data
Source: Zookeys. 2025 Dec 4;1262:175–89. doi: 10.3897/zookeys.1262.164459 (PMC12699342; doi:10.3897/zookeys.1262.164459)
Supplement: Supplementary material 2 — Supplementary figure [file zookeys-1262-175_article-164459__-s002.docx]

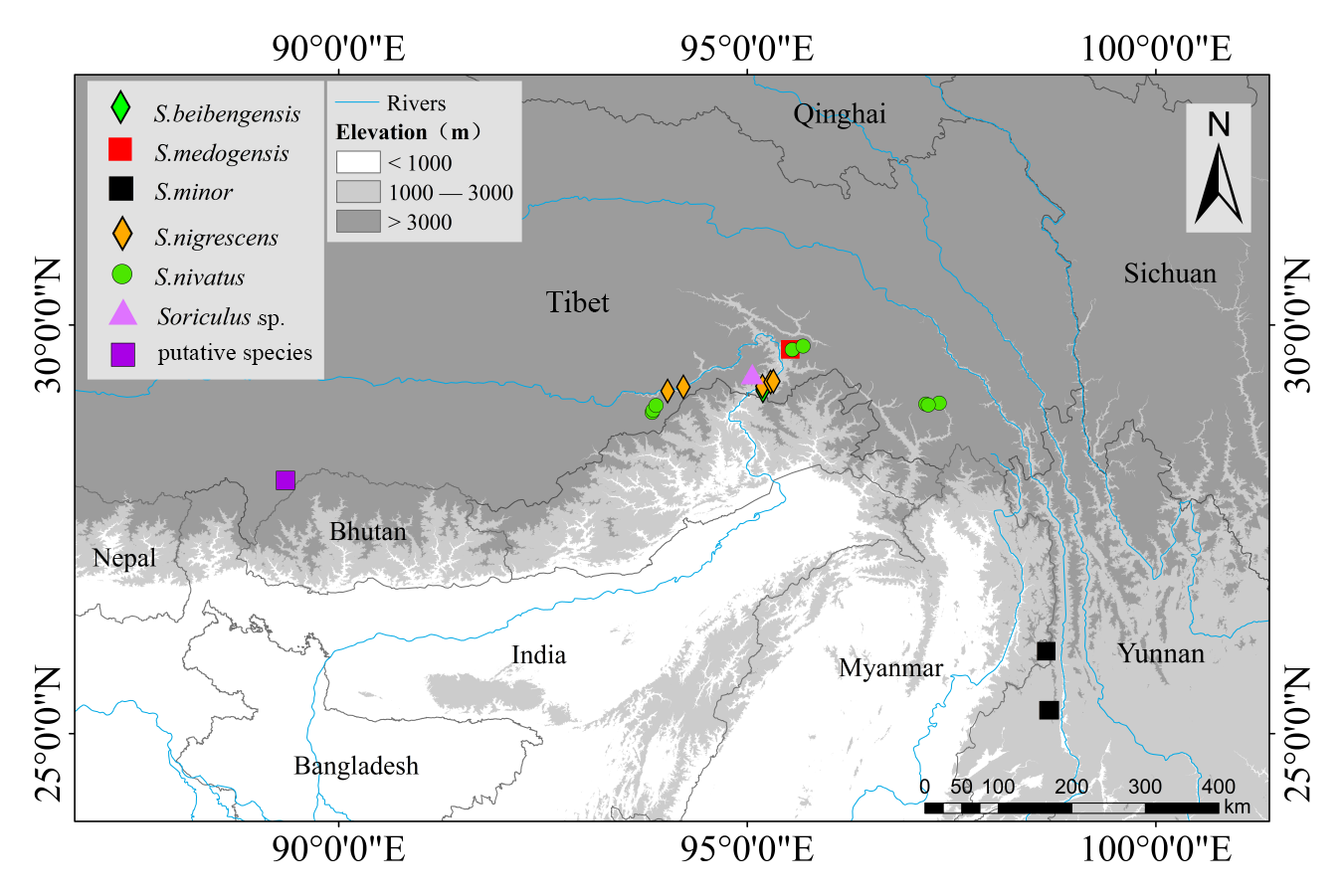
**Fig S1.** Sample localities for the phylogenetic analysis include the putative species (described by Chen et al., 2024), which refers to the Soriculus specimens from Dingri, Tibet, China (voucher csd584; Jiang et al., 2023).

Chen ZZ, Pei XX, Hu JX, Song WY, Khanal L, Li Q, Jiang XL (2024) Multilocus phylogeny and morphological analyses illuminate overlooked diversity of *Soriculus* (Mammalia: Eulipotyphla: Soricidae), with descriptions of two new endemic species from the eastern Himalaya. Zoological Journal of the Linnean Society 201 (2): 534-548. https://doi.org/10.1093/zoolinnean/zlaa172

Jiang HJ, Fu CK, Tang KY, Li FJ, Faiz AUH, Guo KJ, Liu SY, Chen SD (2023) Molecular phylogenetics and diversity of the Himalayan shrew (*Soriculus* nigrescens Gray, 1842) (Eulipotyphla, Soricidae) in Southwest China. Zootaxa 5263 (1): 61-78. https://dio.org/[10.11646/zootaxa.5263.1.3](https://doi.org/10.11646/zootaxa.5263.1.3)
